# The Impact of Liquid Components on Alteration of the Adhesion of Polyacrylate and Silicone Patches

**DOI:** 10.3390/polym15224355

**Published:** 2023-11-08

**Authors:** Bartosz Maciejewski, Joanna Dłabiszewska, Barbara Mikolaszek, Małgorzata Sznitowska

**Affiliations:** 1 Department of Pharmaceutical Technology, Medical University of Gdańsk, Hallera Av. 107, 80-416 Gdańsk, Poland; bartosz.maciejewski@gumed.edu.pl (B.M.); barbara.mikolaszek@gumed.edu.pl (B.M.); 2 Scientific Student Circle “ISPE—Industrial Pharmacy”, Medical University of Gdańsk, Hallera Av. 107, 80-416 Gdańsk, Poland

**Keywords:** patches, pressure-sensitive adhesives, acrylates, silicones, adhesiveness, liquids

## Abstract

Polyacrylates and polysiloxanes are polymers used in pressure-sensitive adhesive (PSA) patches. Liquid additives are co-solvents of the active substances or permeation enhancers, and their compatibility with the polymeric matrix and the effect on adhesive properties should be considered. The patches were prepared from commercial polyacrylates (three types of Duro-Tak^®^) and siloxanes (Bio-PSA^®^ and Soft Skin Adhesive^®^). Propylene glycol, polyoxyethylene glycol, isopropyl myristate, triacetin, triethyl citrate and silicone oil were added (10% *w*/*w*). Formulations were evaluated microscopically and with a texture analyzer in terms of in vitro adhesiveness and hardness. Only silicone oil was compatible with the silicone matrices. The best compatibility of acrylic PSA was observed with triethyl citrate; one out of three Duro-Tak matrices was incompatible with every additive. In all compositions, the adhesiveness was impaired by the liquid additives. A significant drop in adhesiveness was noted after immersion of the patches in buffer and drying. The probe tack test was considered as the most useful for evaluation of the effect of the liquid additive on adhesiveness, but the results obtained with a spherical and cylindrical probe were contradictory. The structural changes caused by the additives were also demonstrated by a 90° peel test, considered as complementary to the tack test.

## 1. Introduction

Adhesive patches as a form of skin medication have been gaining popularity in recent years. The most recent pharmacopeial monograph of patches [1] includes “Cutaneous patches” and “Transdermal patches”. The former have a local effect by absorption of the active pharmaceutical ingredient (API) to the skin or underlying tissue, to achieve, e.g., an analgesic and anti-inflammatory (diclofenac), antirheumatic (capsaicin) or keratolytic (salicylic acid) effect. Transdermal patches are intended to be absorbed through the skin to the blood and exert a systemic effect. Among them, TTSs (transdermal therapeutic systems) are a special type of formulation, characterized by a controlled, constant rate of API release and absorption.

A critical attribute of the patches is their adhesion to the skin surface [1,2]. Due to the use of a special type of polymers, which are classified as pressure-sensitive adhesives (PSAs), the patches adhere well to the skin by gentle pressure but also are easily peeled off, without causing injury to the skin.

For around three decades, the most commonly used PSA polymers have been polyacrylates [3]. They are commercially known as Duro-Tak^®^ [4] and can be either acrylate-vinyl acetate copolymers or plain polyacrylic acid dissolved in ethyl acrylate with the optional presence of other components (isopropanol, toluene or acetylacetone). The three types of Duro-Tak^®^ polymers used in the present study (see Table 1) differ from one another mainly in the content of functional groups (hydroxyl, carboxyl) or type of solvents used, as well as in viscosity and percentage of solid content (33.5–50.5%). The acrylic adhesive films are formed by casting and a solvent evaporation method.

In contrast, silicone (polysiloxanes) PSA films are mainly prepared through condensation reaction of a silanol end-blocked polydimethylsiloxane (PDMS) with a silicate resin. By alteration of the polymer:resin ratio, various properties of the product are achieved—generally, with the increased fraction of the silicone polymer comes increased adhesion [5]. The present study utilizes two types of silicone PSA: Bio-PSA 7-4502^®^ and SSA-MG 7-9850^®^ (see Table 1).While the first one is a solvent-based single-component mass with low viscosity (ca. 650 mPa × s), the latter is a two-component Pt-catalyzed polymer, with each component having ca. 2900 mPa × s viscosity. The adhesive Bio-PSA films are prepared by evaporation of the solvent (ethyl acetate), while the adhesive layers from SSA-MG are formed by polymerization/cross-linking in situ.

An important difference between acrylic and silicone PSA is the more hydrophilic character of the first, while, due to the lack of hydroxyl groups, silicone films are more hydrophobic, which results in very limited solubility of the hydrophilic excipients or API. However, the advantage of silicones is high permeability to oxygen [3].

Considering the patch design, there are two popular types of the formulations: two-layer patches (backing layer and adhesive matrix with API) and three-layer patches (backing layer, non-adhesive matrix with API and adhesive thin layer). The first type (the so-called DiA, drug-in-adhesive) is easier to produce—the PSA layer with API is cast on the backing layer, made of polyethylene or polyester.

In DiA patches, there are two factors responsible for the adhesion: the type of polymer forming the matrix, and the amount and type of API or other components [6]. Liquid additives like propylene glycol or isopropyl myristate can serve as solvents or levigating agents for API but at the same time may have other functions as plasticizers, absorption promoters or crystallization inhibitors. However, the introduction of an additional liquid component may cause physicochemical changes in the PSA layer that can lead to a decrease in adhesiveness. The significance of these changes may depend on the concentration of the additives [7].

The studies described until now focus relatively little on the assessment of the effect of liquid additives on patch adhesive properties. However, it is important to understand that, in addition to biopharmaceutical properties (drug release and absorption) [8,9], mechanical properties of the patch, including adhesion, are equally important. Appropriate manipulation of both the type of liquid additive and its concentration is therefore a priority in order to find formulations with optimal properties.

The choice of a polymer for transdermal medicated patch is a challenge since not only technological but also biopharmaceutical and application issues must be considered. The lack of detailed characteristics of the available PSA polymers, especially in the presence of different additives, results in time-consuming experimental work when transdermal patches are under development. Our long experience in this field shows that the manufacturers of the components for such polymeric matrices do not provide the required information as a starting point for product development. This is why we decided to deeply characterize these matrices with the most common additives.

The work is therefore intended to assess the compatibility of liquid additives with common PSA matrices and to evaluate the exerted changes in their mechanical properties. The methodology of measuring patch adhesiveness that is present in the literature [10,11] focuses mostly on measurement of tackiness (instant adhesion of probe tip to the surface of the patch) or measurement of the force required to peel off a patch from a substrate (usually a stainless steel surface). These methods offer ease of instrumentation; however, they consider slightly different factors, which impacts the obtained results. In our study, the attempt was also made to propose the most suitable method for evaluation of the adhesive properties of the DiA patches.

**Table 1:** Characteristics of the commercial polymers used for the preparation of adhesive matrices [12].

PSA Type	Acrylate			Silicone	
Symbol	A1	A2	A3	S1	S2
Brand name	DuroTak^®^ 387-2287	DuroTak^®^ 87-4098	DuroTak^®^ 87-2852	Bio-PSA MD7-4502	Soft Skin Adhesive MG 7-9850
Structure and chemical name	Acrylate-vinylacetate		Acrylic acid	Polydimethylsiloxanes	
Film formation	Solvent evaporation		Solvent evaporation and crosslinking	Solvent evaporation	Crosslinking
Solvent	Ethyl acetate	Ethyl acetate	Ethyl acetate, n-heptan, isopropanol, toluen, ace-tylacetone	Ethyl acetate	--
Viscosity * (mPa•s)	18,000	6500	2500	1500	2900 **

* Viscosity of a polymer solution. ** Viscosity of part A and part B before the curing process.

## 2. Materials and Methods

### 2.1. Materials

The following materials were used in the study: Acrylate PSA: DURO-TAK 387-2287 (used in A1 formulations), DURO-TAK 87-4098 (A2), DURO-TAK 87-2852 (A3); polysiloxanes: BioPSA 7-4502 (S1) and SSA-MG 7-9850 (S2)—from Dow Corning (Wiesbaden, Germany). The materials are described in Table 1. The following liquid excipients were investigated: triethyl citrate—TEC (Fluka Analytical, Steinheim, Germany), propylene glycol—GP (Avantor Performance Materials, Gliwice, Poland), silicone oil—SO, viscosity 350 cSt (Q7-9120 Silicone fluid 350 cst, Dow Corning, MI, USA), triacetin—TA, polyethylene glycol—PEG 300, isopropyl mirystate—MIP (Sigma-Aldrich, Steinheim, Germany). Polyethylene (PE) film sheet (Esselte, Warsaw, Poland) was used as a backing (supporting) layer for acrylates and Bio-PSA, and Gumosil AD-1 (Silikony Polskie, Nowa Sarzyna, Poland) for SSA.

### 2.2. Methods

#### 2.2.1. Manufacturing of Adhesive Patches

**Figure 1:**
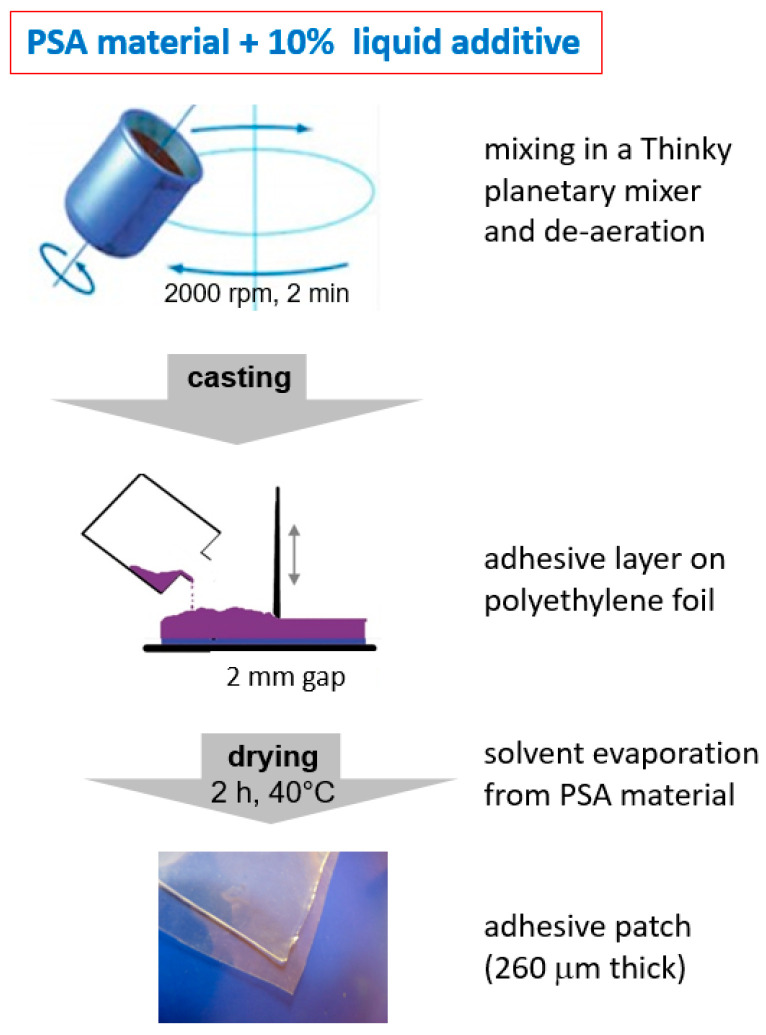
Procedure used for preparation of the adhesive patches from acrylic (A1, A2, A3) and silicone (S1) polymers.

The patches with acrylic PSA matrix (A1, A2, A3) were made using a casting technique described in the literature [13], with slight modification. Figure 1 represents the steps of the procedure. First, liquid additives were mixed with the commercial polymeric substance. The amount of the additive was calculated as 10% *w*/*w* of dry mass of the polymer product. The mixture was prepared in a Thinky ARE-250 planetary mixer (Thinky, Laguna Hills, CA, USA), applying a total of 5 mixing cycles: 30 s of premixing at 500 rpm, then 2 cycles of mixing at 2000 rpm and 2 cycles at 2000 rpm in degassing mode. Finally, the mixture was de-aerated in a high vacuum and cast onto a glass plate covered with PE foil using a Camag TLC plate coater (Camag, Muttenz, Switzerland) to form a film of 2000 µm in thickness. Afterwards, the films were placed for 2 h at 40 °C for solvent evaporation, which was followed by conditioning at room temperature for at least 24 h. The final thickness of the patches was evaluated using a MiniTest 700 magnetic-induction thickness analyzer (Electro Physik, Koln, Germany). This final parameter was strongly dependent on the liquid excipient used, and the value was 265 ± 50 µm on average.

In the same manner, S1 patches were prepared, but the casted layer was 1000 µm thick, which resulted, depending on the composition, in the final thickness of the films being 325 ± 150 µm.

The patches with SSA-MG (S2 compositions) were prepared in a different way. The SSA product consists of two components (A and B), and mixing these two parts at the ratio 1:1 induces the formation of a crosslinked structure. Component “A” and the liquid excipient were weighed directly in the container dedicated for use with the Thinky mixer. The mixture was then processed in cycles: 30 s at 500 rpm, 2 min at 2000 rpm, and 2 min at 2000 rpm in de-aeration mode. Afterwards, component “B” was added, and mixing was performed using a cooling adapter to slow down the solidification process. The prepared mass was cast on a previously prepared layer of Gumosil AD-1 and distributed with a Camag TLC plate coater to form a film of 800 µm thickness. Finally, the patches were conditioned at room temperature for 24 h to finish the polymerization.

#### 2.2.2. Microscopic Analysis

The adhesive patches were investigated using a Nikon Eclipse 50i optical microscope (Nikon, Tokyo, Japan). The samples were observed in transmitted light to search for incompatibilities or structural flaws (phase separation, crystallization, etc.).

The SEM evaluation was performed with a Phenom Pro scanning electron microscope (Phenom World, Eindhoven, Netherlands) using a backscattered electron detector (BSD) and a secondary electron detector (SED). Samples of the patches were sputtered with gold prior to investigation.

#### 2.2.3. Hardness

Measurements of hardness were conducted with a TA.XT Plus texture analyzer (Stable Micro Systems, Godalming, UK) equipped with a conical Perspex probe. Since the adhesive layer in the patches was too thin for the measurement, special samples for this test were prepared by casting in a flat vial a small amount of each film-forming composition to obtain sufficient thickness (at least 5 mm in the dry state), without a backing layer. The hardness was measured as a force needed to penetrate the sample to the depth of 2.0 mm. The test parameters are presented in Table 2.

#### 2.2.4. Probe Tack Test

**Figure 2:**
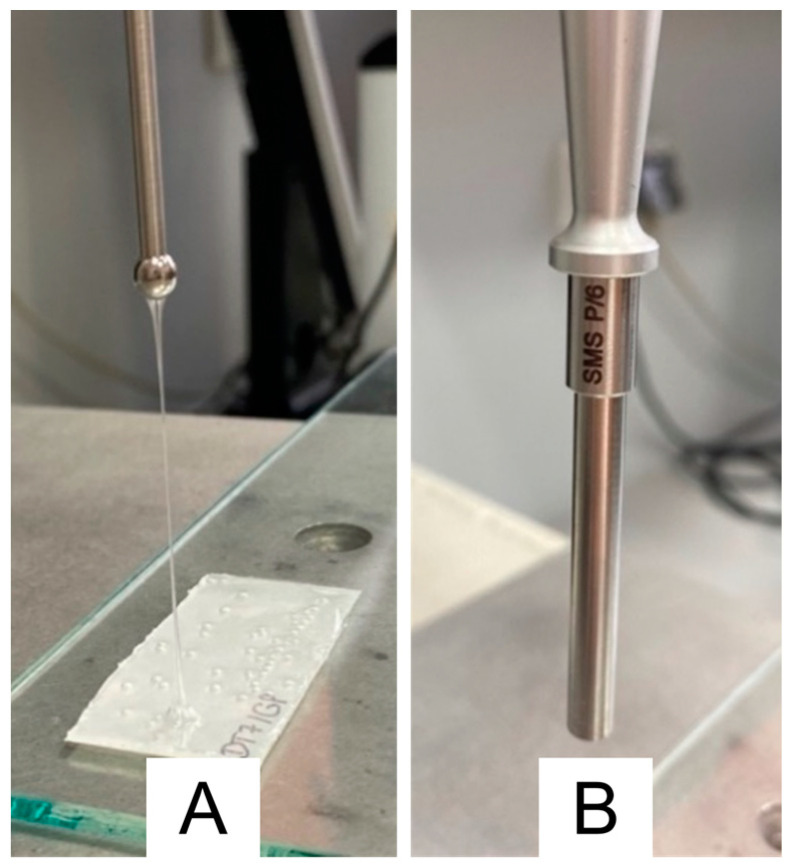
Probes used in the tack test: (**A**)—spherical probe (P5S) in contact with the A1 patch, (**B**)—cylindrical probe (C6).

The test was performed with a TA.XT Plus texture analyzer equipped with two types of probes: Ø 5 mm spherical (P5S) and Ø 6 mm cylindrical (C6). The probes are shown in Figure 2.

The patch samples with area of ca. 6 cm^2^ were fixed to a glass plate with double-sided adhesive tape. The measured parameter was tackiness (adhesiveness, instantaneous adhesion) expressed as the force (N) required to separate the probe from the surface of adhesive patch at 10 mm/s after contact with 4 g force for 2 s (Table 1). The measurements were performed in 6 various areas of the sample.

#### 2.2.5. Peel Test

**Figure 3:**
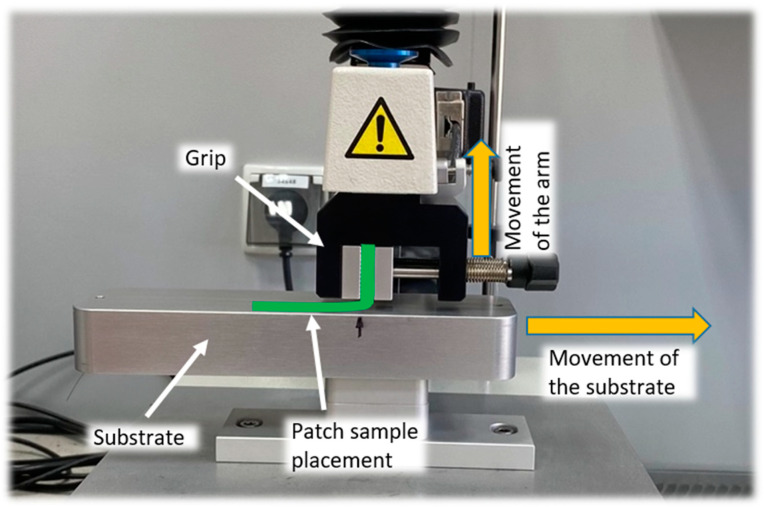
The setup for the 90° peel test with dedicated attachment to texture analyzer.

The test was performed according to the USP method for the “Peel adhesion test” described in the monograph “Topical and transdermal drug products—product quality tests” [14]. The scheme of the test setup is presented in Figure 3, and the test parameters are presented in Table 1. Each sample (1 cm × 6 cm) was attached to the flat steel surface (a substrate) with double-sided adhesive tape and pressed with a 1 kg weight for 1 min. One end of the patch (ca. 1.5 cm) was attached to the arm of the texture analyzer and peeled from the substrate under a 90° angle. Based on the acquired force–distance curve, the initial peel force and mean peel force were determined and evaluated.

#### 2.2.6. Influence of Swelling and Erosion on Tack Properties of the Patches

**Figure 4:**
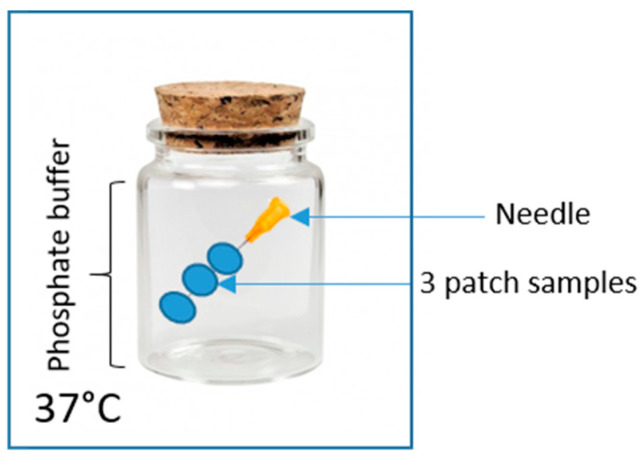
Scheme of the swelling test setup.

Selected formulations were subjected to the swelling/erosion analysis. For this purpose, 3 round samples (area 0.73 cm^2^) were cut out of the investigated patches. The samples were placed on a needle (acting as a weight to prevent floating; see Figure 4), submersed in 60 mL of pH 5.6 phosphate buffer, and kept at 37 °C for 96 h (or until reaching a plateau in weight increase, but not longer than 7 days), after which the wet (swollen) mass was evaluated. Afterwards, the patch samples were subjected to the tack test using the C6 probe. Then, the samples were dried at 37 °C for 48 h, and their mass was again measured. For the dried samples, the tackiness was evaluated again using the C6 probe.

#### 2.2.7. Statistical Analysis

The statistical analysis of the obtained results was performed with Statistica 13 software (Tibco software, Palo Alto, CA, USA) using ANOVA with the RIR Tukey post hoc test.

## 3. Results and Discussion

### 3.1. Visual Assessment

Visual observation was performed for each produced patch. This approach allowed early detection of incompatibilities and potential or actual problems with performing measurements. It was observed that all formulations, except A1, were formed into a coherent adhesive layer. All A1 compositions, however, showed incompatibility with utilized liquid additives. In contrast to the plain patch, without additives, the samples did not fully solidify upon solvent evaporation, and at the investigated levels of liquid excipients, A1 formulations showed very low cohesiveness. That led to behavior similar to viscous liquid—the probe during the tack test was pulling “strains” of patch structure and was unable to detach from the probe (see Figure 2A). Therefore, it was impossible to determine the tackiness. Because the main point of the following work was to evaluate and compare the impact of various additives used at the fixed concentrations of 10% *w*/*w*, instead of adjusting the polymer to excipient ratio, the A1 matrix was excluded from further investigation.

In silicone patches, an outcome similar to A1 compositions was observed in the S1 patch with MIP (S1-MIP). Even when the amount of MIP was lowered (to 5% or to 2%), the creep behavior of the patches affected the measurement of mechanical properties; therefore, the results are not presented in this work.

### 3.2. Optical Microscopy and SEM

**Figure 5:**
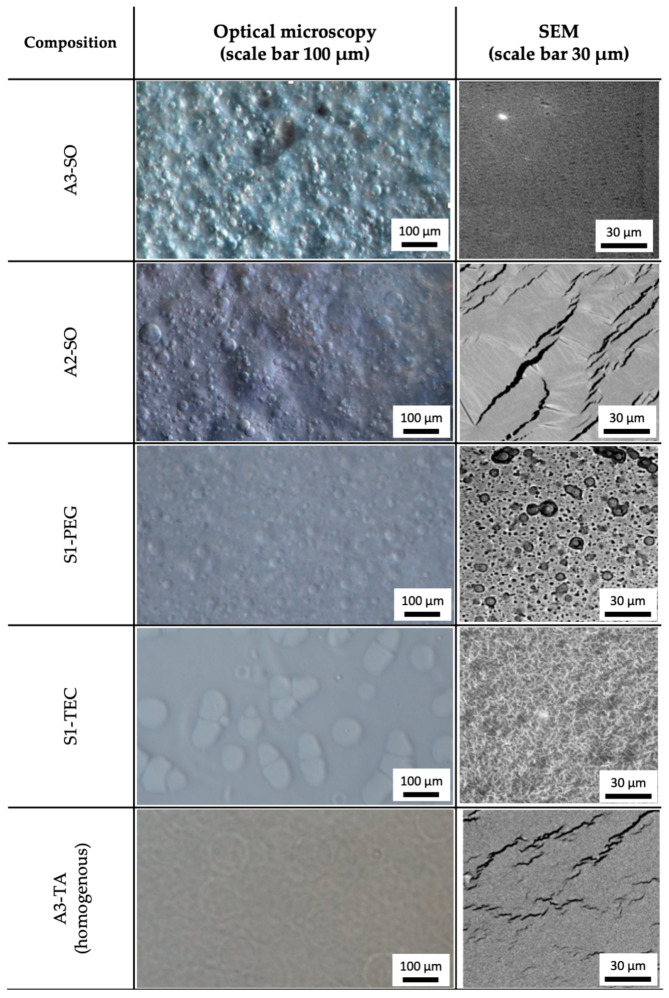
Sample images of incompatibilities in acrylate and silicone patches: droplets of the liquid additives are dispersed in the polymeric matrix, which is visualized by optical microscopy but not seen when the surface of the patch is observed with SEM. A sample of a visually homogenous structure (A3-TA) is also presented.

Microscopic analysis of acrylic formulations showed good compatibility (no signs of phase separation) in the majority of samples, except for acrylate matrices containing silicone oil (A3-SO and A2-SO) and silicone matrices with PEG and TEC (S1-PEG and S1-TEC). On the other hand, the silicone-based formulations showed very limited compatibility, and phase separation was present in patches with TEC, TA, PEG, and GP for S2 compositions, and in patches with PEG and TEC for S1 compositions. The images showing incompatibilities in these formulations are shown in Figure 5. Although the incompatibilities were visible under the optical microscope, they did not impact the patch structure visible with the SEM technique, when gold sputtering was employed at the sample preparation step. It is possible that sputtering the patches with gold prior to SEM analysis successfully masked the symptoms of phase separation.

As expected, the results indicate that strongly hydrophobic silicone oil does not form a homogenous mixture with more hydrophilic acrylic matrices; however, the compatibility with other additives is unpredictable, depending on the particular PSA compositions: for example, hydrophilic GP can be added at concentration of 10% *w*/*w* to A2, A3 and S1 but not to A1 or S2. The difference between S1 and S2 can be explained by easier incorporation of GP when it forms the liquid phase with ethyl acetate present in the S1 material.

### 3.3. Adhesive Properties

Adhesive properties of the investigated patch formulations were evaluated, as indicated in Section 2.2.4 and Section 2.2.5, using the probe tack test and 90° peel test.

#### 3.3.1. Probe Tack Test

The tack property is a measure of an initial bonding of adhesive patch to the substrate, with minimum pressure applied. The tack value is based on the actual molecular interaction between the two interacting surfaces. Being impacted by both viscoelastic properties of the investigated patch and the free surface energies of the adhesive and substrate, the probe tack test, first introduced in 1999 [15], is currently one of the most popular tests used in assessment of adhesiveness of the dermal patches [11,16,17].

An important feature affecting the measured tackiness is a probe geometry. The most often used are spherical (round) and cylindrical (flat) probes. The difference between them lies in the curvature of the tip and in the contact surface area—which impacts the deformation pattern of a viscoelastic material being pulled by the probe in the tack test [18]. In our work, both probe geometries were utilized, with the attempt to choose the most suitable model.

**Figure 6:**
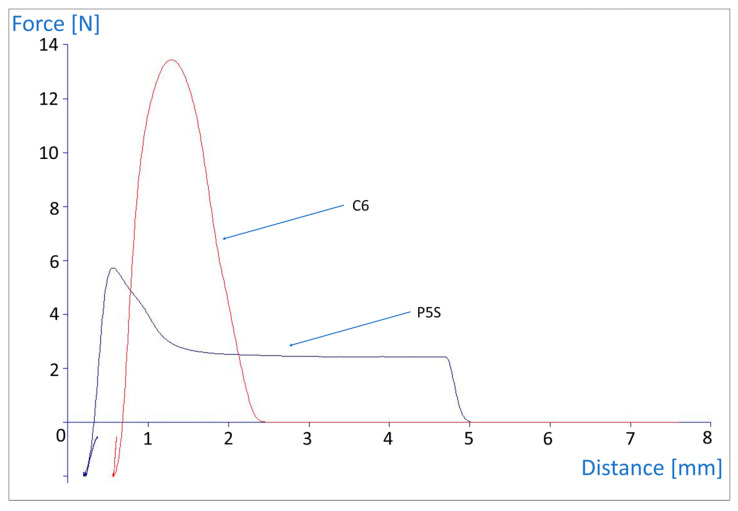
Comparison of the results collected with a spherical (PS5) and cylindrical (C5) probe in the tack test for the A2-TA patch.

The measurements with the C6 probe resulted in higher values of both detachment force and work of adhesion. This was to be expected due to the higher probe–patch contact surface (ca. 29.6 mm^2^) than in case of a spherical P5S. The exemplary tackiness graphs obtained for the same composition (A2-TA) with both probe geometries are shown in Figure 6. The differences between measurements performed with C6 or P5S probes are clearly visible also in plain polymer patches, without liquid additives, as shown in Table 3.

**Figure 7:**
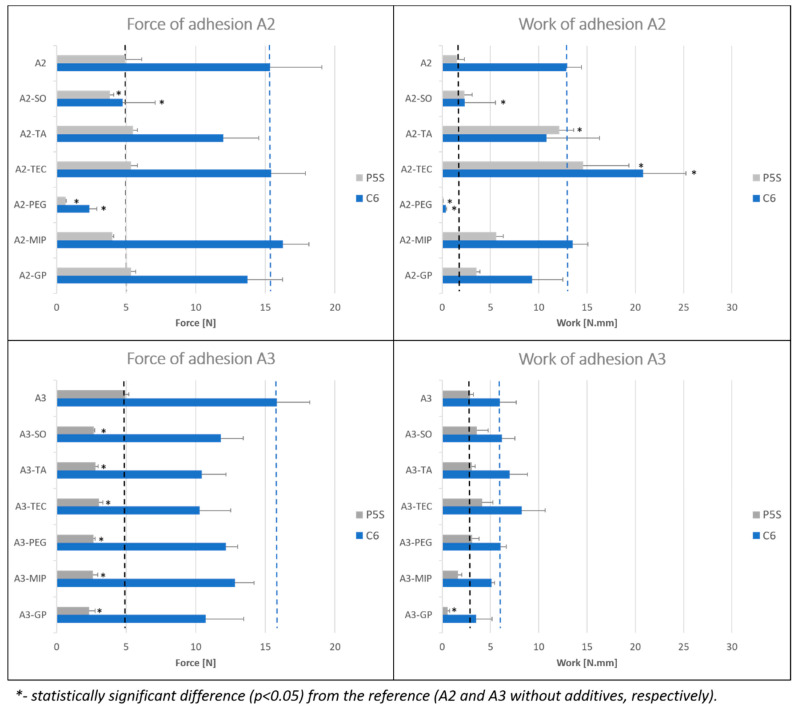
Results of probe tack test (force and work of adhesion) in acrylate PSA formulations: A2 and A3 with liquid additives (average ± SD; n = 10). P5S—spherical probe, C6—cylindrical probe. The reference values (respective patches without liquid additives) are marked as dashed lines.

**Figure 8:**
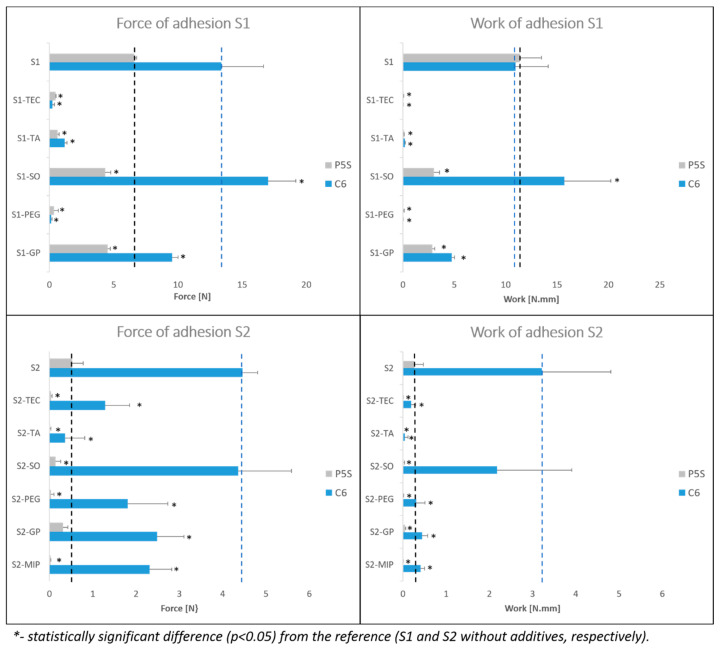
Results of probe tack test in silicone PSA formulations: S1 and S2 with liquid additives (average ± SD; n = 10). P5S—spherical probe, C6—cylindrical probe. The reference values (respective patches without liquid additives) are marked as dashed lines.

Figure 7 and Figure 8 show the results obtained for all investigated patches: the tackiness, expressed as maximum detachment force (N) and, work of adhesion, calculated as area under the force–distance curve.

In A3 compositions, both probes revealed a slight decrease in the adhesion force in the presence of all liquid components, and the effect was similar for all tested substances (Figure 7). Only small changes were observed in the work of adhesion values, with the significant decrease (*p* < 0.05) of this parameter in the formulation containing GP.

In A2 compositions, a significant decrease of both detachment force and work of adhesion was observed only in patches with PEG and SO; however, in the latter case, this was evident only when the C6 probe was used. Since the low compatibility of A2 and SO was observed (see Section 3.2 above), it is possible that the high contact area in the C6 probe included a relatively high amount of SO droplets on the surface of the patch and thus resulted in the lowering of measured values. Due to the phase separation effect, which can potentially negatively influence the stability and biopharmaceutical properties of the patches, the compositions A3-SO and A2-SO were excluded from further investigation.

In A2-TA and A2-TEC the liquid excipient had no effect on the detachment force, although the work of adhesion was significantly increased. That can be explained by a significant plasticizing effect; however, it has been reported that the work of adhesion can be considerably increased by introduction of oxygen-carrying chemicals to the PSA polymer [19].

According to the results obtained with a spherical PS5 probe in the S1 and S2 formulations, all liquid excipients caused significant or even almost complete loss of adhesiveness. However, the results from the experiments with the C6 probe show that SO was an exception, and in the case of the S1 formulation, this additive even caused an increase of adhesiveness. Such a discrepancy requires further investigation of in vitro–in vivo correlation. Our preliminary in vivo study showed that SO in both silicone matrices did not affect negatively the adhesiveness of the patches, which is in favor of the C6 probe, although this should be confirmed in larger study. It is also important to note that in the peel test, described below, the mean force determined was higher for the S1-SO patch than for the S1 patch.

In summary, it is clear that liquid excipients exert a high impact on the adhesive properties of the patches, both the acrylate and silicone type. In all cases, the liquid excipients diminish the in vitro force of adhesion, whereas in certain cases the reduction in adhesive properties can be substantial, even if the liquid excipient is apparently compatible with PSA. It is also important to note that acrylate PSAs display overall higher tackiness than silicones.

#### 3.3.2. Peel Test

**Figure 9:**
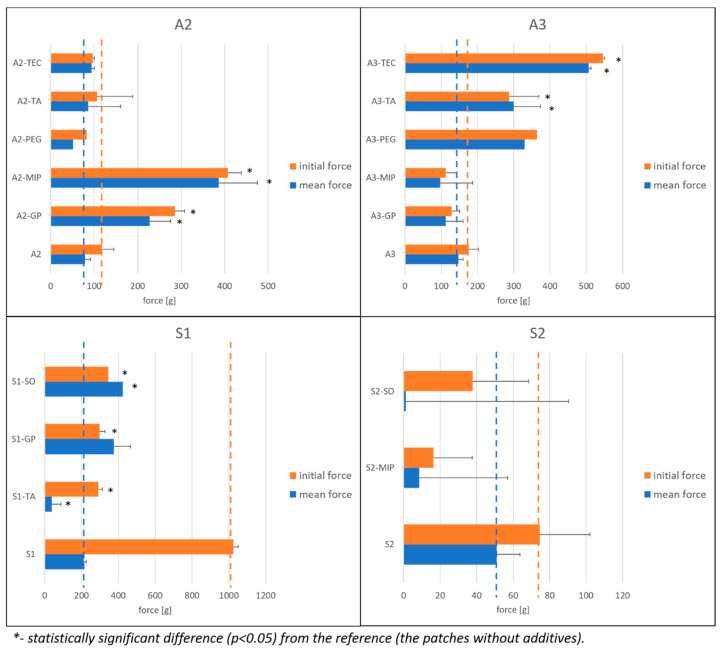
Results of the peel test for acrylate (A2, A3) and silicone (S1, S2) patches—the effect of liquid additives (acrylates: n = 6, except for A3 where n = 3; S1: n = 5, S2 n = 3). The reference values (blank patch without liquid additives) are marked with dashed lines.

The peel adhesion test evaluates the force required to peel off an adhesive from a surface of a substrate at a certain angle. Usually stainless steel substrates and 90° or 180° angles are used [10], which is compliant with USP chapter “Topical and transdermal drug products—product quality tests” [14]. The peel adhesion is also dependent on the dwell time and pressure applied upon attachment of the patch to the substrate. The parameters of the tests performed are compliant with USP and are presented in Section 2.2.5. The tests were performed for selected formulations, and the results are presented in Figure 9. By analyzing the graphs, one can observe that, in case of some samples, the initial peel force is substantially higher than the mean peel force. This was especially evident in the case of the S1 patch, without additives. Such an effect is difficult to explain due to little or no literature on the subject.

It is clearly visible that silicone- and acrylate-based patches exert entirely different peel properties. Moreover, in acrylate patches, the effect of liquid additives was strongly dependent on the type of PSA: in the A2 matrix, the increased initial and mean peel forces (with the maximum obtained values up to 600 g) were observed for A2-MIP and A2-GP, while these additives had no effect in A3 patches; in this case, TEC, TA and PEG produced stronger interaction. At the same time, the plain S1 matrix shows disproportional values of initial (ca. 1000 g) and mean (ca. 200 g) peel force. It is important to note that in the preliminary in vivo study, the S1 matrix was described as painful upon removal from the skin, while no such disadvantage was found for the S2 patch.

In the S1 patch, the liquid additive TA appears to significantly decrease both the initial and mean peel force, while SO and GP cause an increase in the mean peel force. On the other hand, in S2, both tested liquid additives (SO and MIP) cause a substantial drop in peel forces; however, the significance of the measured values is questionable due to very high standard deviations.

**Figure 10:**
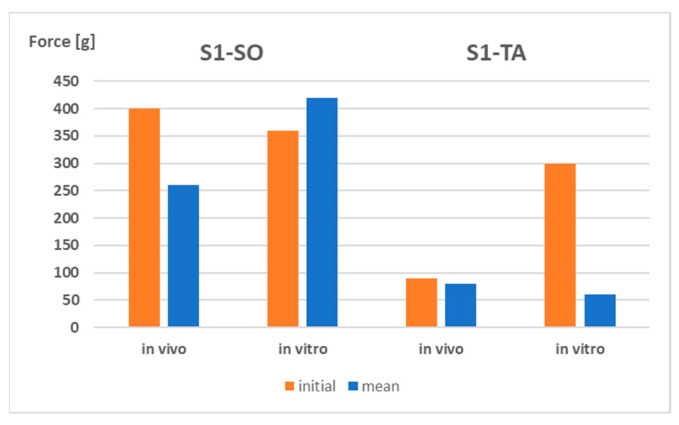
The 90° peel test for silicone patches S1-SO and S1-TA—comparison of the results obtained in vitro and in vivo.

Preliminary results of the test performed on the S1 patches in vivo confirmed that TA, in comparison with SO, decreased the adhesiveness of the film substantially (Figure 10).

Although the tackiness (results described in Section 3.3.1) of the A3-based compositions was slightly reduced by all of the used liquid excipients, a similar effect in the peel test can be observed only in case of the addition of MIP and GP. However, due to high deviations in single measurements, many of the differences cannot be classified as statistically significant.

Contrary to the A2 compositions, in the A3-based formulations there is a noticeable impact of TEC and TA on both the initial and mean peel force. What is more, the addition of MIP, which caused a substantial increase in the peel force in the A2 composition, in A3 resulted in a slight reduction of the parameter; however, the forces are not significantly different from the reference samples. There is also an increase in peel force in the case of the A3-PEG composition; however, due to limited testing material, the measurement was performed in single run only, and therefore the recorded difference from the plain A3 patch is in this case questionable.

As already mentioned, PSA films are characterized by good adhesiveness under small pressure and at the same time by easy detachment without disturbing the integrity of the skin layer—the stratum corneum. One can consider the results of the peel test as more useful for the evaluation of the forces during detachment of the formulation from the skin, while the tack test indicates the strength of the adherence, without the peeling procedure. Although there is no such clear interpretation of these tests in the literature, it is not surprising that the tests can give opposite results; for example, MIP does not change the tackiness of the A2 matrix, but higher force is required to remove it from the site of application.

Overall, depending on the matrix type, the liquid additives have different effects regarding the force required to peel off the patch from the substrate. Although this effect is unpredictable, especially for the in vivo situation, the results of the performed studies indicate that the choice of additive can ameliorate too-strong or too-weak adherence of the patch to the skin.

### 3.4. Hardness

**Figure 11:**
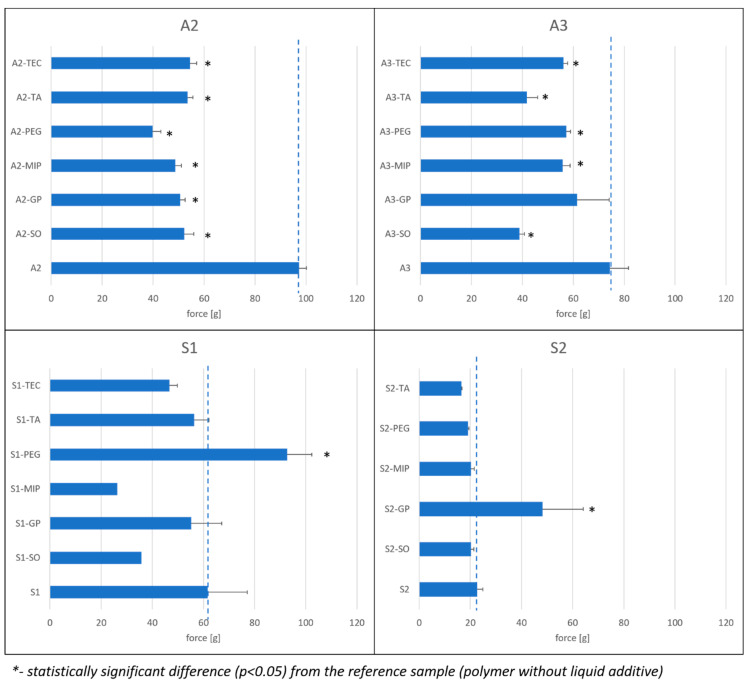
Hardness of acrylate (A2, A3) and silicone (S1, S2) matrices—the effect of liquid additives (n = 6, except for S1-MIP: n = 2 and S2-GP: n = 3). The reference values (for the respective patch without liquid additives) are marked with dashed line.

The liquid additives to the PSA compositions can exert an effect on the structural integrity of resulting patches, which can be expressed by measurement of hardness (the force required to penetrate the patch). The measurements were performed according to the procedure described in Section 2.2.3. The hardness values measured for all investigated acrylate and silicone compositions are presented in Figure 11.

In A2 and A3 compositions, it was observed that each investigated liquid excipient resulted in the lowering of patch hardness; however, the differences are less pronounced in A3 formulations, with GP having the smallest effect (not statistically significant).

The measurements performed on S1 and S2 samples showed that only addition of PEG or GP, respectively, significantly alters the samples by increasing hardness. It is, however, noteworthy that these compositions show incompatibility between the polymer and the additives (see Section 3.2). It is also peculiar that S1-TEC shows phase separation (Figure 5), which apparently has only a small effect on the hardness of the patch.

In summary, in acrylate compositions, the liquid excipients caused a reduction in hardness of the polymer structure, as was expected due to the plasticizing effect of the liquids. However, the effect was more pronounced for the A2 matrix. In silicones, there is no clear correlation between the observed incompatibilities and changes in the structure of the polymer matrix measured as hardness.

### 3.5. Swelling and Erosion

When patches are applied to the skin, the adhesive layer is subjected to water from sweat glands and from transepidermal water loss. Some patches, like those for scar treatment, can be used for several days, with a recommendation to wash them between applications. A simulation of such a situation can be performed by measurement of the water absorption rate upon submersion of the patch in the pH 5.6 phosphate buffer (pH value similar to that of a skin). The investigation of changes in mass and tackiness upon submersion was performed according to the description presented in Section 2.2.6.

**Figure 12:**
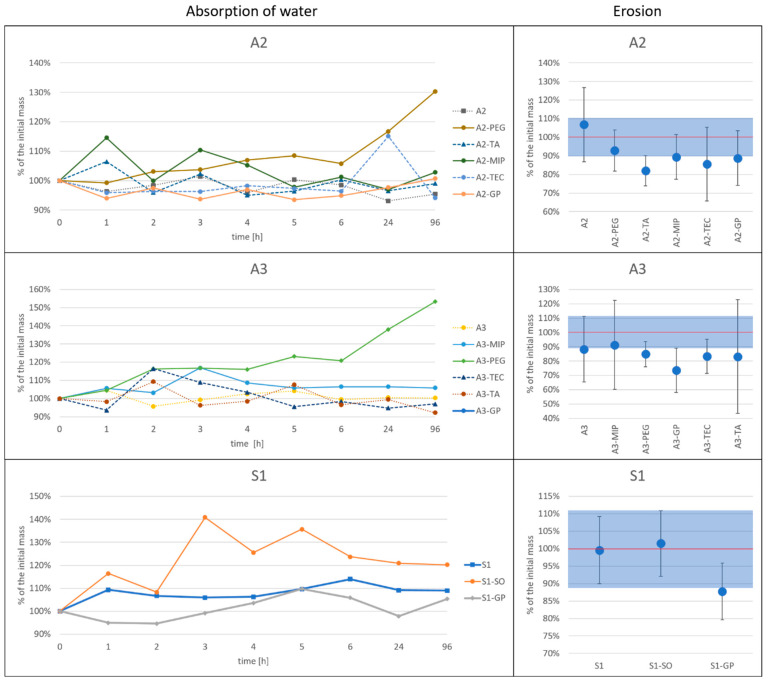
Change in the mass (absorption of water) during submersion of the patches in phosphate buffer for 96 h and the residue mass after drying (erosion – the initial mass is marked with red line and the limit ±10% is marked with a blue area).

Due to the small size of the samples (ca. 30–70 mg of PSA), the measurements of the small increase in the weight were not sufficiently reproducible—even in the equilibrium phase, the recorded masses varied in some samples by less than 10% from the dry mass (Figure 12). Considering this, the conclusion can be drawn that no significant absorption of water was observed either for the more hydrophilic A2 or A3 patches or for the more hydrophobic S1 patch. Larger changes occurred in A2 and A3 patches with PEG and in S1 with SO, but the effect does not correlate with the physicochemical character of these additives.

Figure 12 presents not only the changes in the mass of samples during submersion in the buffer, but also the mass residue after drying the wet sample. The latter indicates the dissolved/eroded portion of each formulation.

The mass of the samples dried after submersion in the buffer solution for 96 h was essentially similar to the initial mass of the samples, which means that actual erosion (rinsing out/dissolving parts of the patch) did not occur at a noticeable level. The highest mass reduction was observed in the A3-GP formulation, in which the residual mass after swelling and drying was around 75% (±15%) of the initial mass. However, in general, there was no clear relationship between erosion and the aqueous solubility of the additives. These observations are important when dissolution profiles of API from such types of matrices are analyzed.

The water absorption and eventual erosion can potentially impact the adhesive properties of the patch. Therefore, to assess the possibilities of adhesiveness alterations upon application of the patch on the skin, the samples from the swelling test were subjected to the probe tack test, with use of the C6 probe. The results of the tests performed for both the acrylate and silicone patches are presented in Table 4.

As expected, the moisture present in the patches, even in the amount up to 10%, reduces the initial tackiness of the patches. Although the smallest effect was observed for the acrylic patches with TA, it is still unclear how much the effect depends on the type of a liquid additive.

In almost all the tested samples, a recovery of tackiness was observed after drying. However, in no case, both in acrylic or silicone patches, was the initial adhesiveness restored. The exceptions to this were compositions A2-TA and A2-MIP, where drying the swollen samples caused a further drop in tackiness, from 48.8 to 22.9% and from 21.1 to 7.2% of the initial value, respectively.

From the results presented above, one can conclude that the absorption of water in the films is very small and unrelated to the hydrophilicity of the matrix or liquid additives, but it has a noticeable negative impact on the adhesiveness of the patches.

## 4. Conclusions

The presented work focused on investigating the compatibility of the most commonly used liquid excipients with exemplary PSA acrylate and silicone matrices by measuring their adhesive properties. The study showed that higher compatibility with liquid excipients and better adhesive properties were exhibited by the patches based on acrylates. However, this was true only for two out of the three tested acrylic PSAs, as type A1 polymer was incompatible with all liquid additives, which rendered testing of these compositions impossible. It is crucial to note that even if the liquid excipient is apparently compatible with PSA, the adhesive properties can still be significantly reduced.

The results of this work clearly indicate that liquid excipients exert a high but also unpredictable impact on the adhesive properties of the patches, both the acrylate and silicone types. Overall, the results of the performed analyses show that acrylate matrices are more versatile and better performing in adhesion tests; however, this also depends on the type of the acrylic PSA used. The liquid additives did not significantly change the water uptake by the patches immersed in a buffer; the absorption of water was very low in both silicone and acrylic patches. However, after being in contact with water, the patches showed a significant loss of their adhesive properties.

The characteristics of the mechanical properties of the adhesive patches greatly depends on the experimental model used. There is no satisfying correlation between the results of adhesiveness measured with probes of different geometries. In addition to the tack test, the peeling test should be used to determine how easily the patches adhere to the surface and the strength of the adherence. The two tests should not be considered interchangeable due to the fact that they consider different factors impacting the overall adhesive performance of the investigated patches. Therefore, the results from these two tests should not be directly compared; instead, they should be used to supplement each other to obtain a wider range of information regarding the properties of the adhesive patches. Certainly, it must be also taken into consideration that the metal substrate used in vitro does not allow accurate determination of the in vivo behavior of the patches.

## Figures and Tables

**Table 2:** The parameters of the tests performed with a texture analyzer: hardness, probe tack and peel test.

Parameter		Hardness	Probe Tack	90° Peel Test
Active distance		2.0 mm	-	4.5 cm
Probe speed	Pre-Test	0.1 mm/s	1.0 mm/s	1.0 mm/s
	During the test	1.0 mm/s	10.0 mm/s	5.0 mm/s
	Post-Test	10.0 mm/s		10.0 mm/s
Maximum force/Detection limit		5.0 g	-	5.0 g
Contact force		-	4.0 g	-
Time of contact		-	2.0 s	-
Temperature		22 ± 2 °C		
Relative humidity		45 ± 5%		

**Table 3:** Comparison of adhesion (tackiness) parameters for plain acrylate and silicone patches, without liquid additives, measured with spherical (PS5) and cylindrical (C6) probes (average values, n = 6–10).

Formulation	Force of Adhesion (N)		Work of Adhesion (N•mm)	
	P5S	C6	P5S	C6
A2	4.95	15.33	3.55	9.30
A3	4.95	15.83	2.86	5.95
S1	6.61	13.40	11.39	10.96
S2	0.53	4.46	0.26	3.21

**Table 4:** The effect of liquid additives on the tackiness of swollen and subsequently dried acrylic (A2 and A3) and silicone (S1) patches (S2 compositions were not included in the test).

Formulation	Tackiness (Detachment Force, Probe C6)				
	Initial Value (N)	Swollen (N)	(%) of Initial	Swollen + Dried (N)	(%) of Initial
A3	15.82	Not tested		5.83	36.8
A3-PEG	12.15	1.63 *	13.4	2.99 *	24.5
A3-GP	10.70	Not tested		4.47 *	41.8
A3-TEC	10.27	0.86 *	8.4	6.44	62.7
A3-TA	10.44	4.82 *	46.2	5.31	50.8
A3-MIP	12.82	0.26 *	2.0	3.28 *	25.6
A2	15.32	Not tested		7.61 *	49.6
A2-PEG	2.37	Not tested		1.17	49.2
A2-GP	13.72	0.63 *	4.6	8.01 *	58.4
A2-TEC	15.42	0.33 *	2.1	8.49 *	55.1
A2-TA	11.99	5.86 *	48.8	2.74 *	22.9
A2-MIP	16.25	3.43 *	21.1	1.17 *	7.2
S1	13.40	Not tested		5.90	44.0
S1-GP	9.57	Not tested		4.07 *	42.5
S1-SO	17.02	Not tested		6.21 *	13.5

*—statistically significant difference (*p* < 0.05) from initial value (before swelling and/or drying).

## Data Availability

Data supporting this study are available on request.
